# Construction, Validation, and Visualization of Two Web-Based Nomograms to Predict Overall and Cancer-Specific Survival in Patients with Gastric Cancer and Lung Metastases

**DOI:** 10.1155/2021/5495267

**Published:** 2021-11-01

**Authors:** Honghong Zheng, Zhehong Li, Jianjun Li, Shuai Zheng, Enhong Zhao

**Affiliations:** ^1^Department of Gastrointestinal Surgery, Affiliated Hospital of Chengde Medical University, Chengde, China; ^2^Department of Orthopedic, Affiliated Hospital of Chengde Medical University, Chengde, China

## Abstract

**Background:**

The lung is one of the most common sites of metastasis in gastric cancer. Our study developed two nomograms to achieve individualized prediction of overall survival (OS) and cancer-specific survival (CSS) in patients with gastric cancer and lung metastasis (GCLM) to better guide follow-up and planning of subsequent treatment.

**Methods:**

We reviewed data of patients diagnosed with GCLM in the Surveillance, Epidemiology, and End Results (SEER) database from 2010 to 2015. The endpoints of the study were the OS and CSS. We used the “caret” package to randomly divide patients into training and validation cohorts in a 7 : 3 ratio. Multivariate Cox regression analysis was performed using univariate Cox regression analysis to confirm the independent prognostic factors. Afterward, we built the OS and CSS nomograms with the “rms” package. Subsequently, we evaluated the two nomograms through calibration curves, receiver operating characteristic (ROC) curves, and decision curve analysis (DCA). Finally, two web-based nomograms were built on the basis of effective nomograms.

**Results:**

The OS analysis included 640 patients, and the results of the multivariate Cox regression analysis showed that grade, chemotherapy, and liver metastasis were independent prognostic factors for patients with GCLM. The CSS analysis included 524 patients, and the results of the multivariate Cox regression analysis showed that the independent prognostic factors for patients with GCLM were chemotherapy, liver metastasis, marital status, and tumor site. The ROC curves, calibration curves, and DCA revealed favorable predictive power in the OS and CSS nomograms. We created web-based nomograms for OS (https://zhenghh.shinyapps.io/aclmos/) and CSS (https://zhenghh.shinyapps.io/aslmcss/).

**Conclusions:**

We created two web-based nomograms to predict OS and CSS in patients with GCLM. Both web-based nomograms had satisfactory accuracy and clinical usefulness and may help clinicians make individualized treatment decisions for patients.

## 1. Introduction

Gastric cancer (GC) is one of the most common malignant tumors of the gastrointestinal tract, accounting for the third and fifth causes of cancer deaths in men and women worldwide, respectively [1]. According to the 2018 Global Cancer Center statistics [1], there were approximately one million new cases of GC and approximately 780,000 GC-related deaths worldwide. Although radical surgery is currently effective in treating localized GC, recurrence or metastasis still occurs in 25% to 40% of patients after surgery [2–4]. According to relevant studies, the lung is a frequent metastatic organ in patients with GC [5], and the incidence of lung metastasis (LM) after GC surgery ranges from 1.3% to 3.8% [6–10]. Moreover, there is a lack of mature therapy standards for gastric cancer and lung metastasis (GCLM), and the 5-year survival rate of patients with GCLM is <5% [11]. At this stage, few studies have reported prognostic factors regarding the survival of patients with GCLM. Therefore, establishing a prediction model for patients with GCLM is clinically significant.

The treatment of GCLM has been recently diversified [12–15]; however, the poor surgical outcome and complications associated with lung-occupying lesions in patients with GCLM lead to worse prognosis. Kong et al. [16] reported that the median survival of patients with GCLM is only four months. Moreover, studies have shown that the prognostic influences of GCLM generally include tumor histological grade, T stage, concurrent pulmonary metastases, primary lesions not subjected to surgery, bilateral pulmonary metastases, combined extrapulmonary metastases, and chemotherapy [17]. Regrettably, no studies have combined the relevant variables to assess the prognosis of GCLM.

A nomogram is a simple, multivariate visualization tool in oncology for predicting and quantifying individual patient survival, to aid clinical decision-making and promote precision medicine [18–21]. In addition, the web-based nomogram, also known as “predictive probability web page calculator,” is a web page based on Shiny. This nomogram is a product of the electronic era, and the user just has to select the appropriate variable and click “Predict” to draw the probability of occurrence of the corresponding characteristics of patients, which is convenient and more practical [22]. Consequently, we aimed to devise two web-based nomograms to predict the overall survival (OS) and cancer-specific survival (CSS) in patients with GCLM based on the Surveillance, Epidemiology, and End Results (SEER) database.

## 2. Materials and Methods

### 2.1. Data Source and Inclusion Criteria

In this study, our data were obtained by downloading the SEER∗Stat software version 8.3.6. The SEER database is a public database, exempt from medical ethics review, and does not require informed consent. Strict inclusion and exclusion criteria were also developed, and the nadir criteria are listed below. The inclusion criteria were as follows: (I) patients diagnosed with GCLM between 2010 and 2015; (II) demographic variables, including age, race and gender, marital status, and insurance status; and (III) available tumor characteristics, including histological grade, T stage, N stage, brain metastasis, bone metastasis, and liver metastasis. The exclusion criterion was incomplete information. Next, we randomized the patients into training (70%) and validation cohorts (30%). In this study, patients in the training and validation cohorts were used to develop and validate the nomograms, respectively.

### 2.2. Clinicopathological Factors

Clinicopathological factors for the following variables were extracted: age (<60 and ≥60 years), race (white, black, and other), sex (female and male), histologic type (adenocarcinoma, signet ring cel1, intestinal type, other), T stage (T1, T2, T3, and T4), N stage (NO, N1, and N3), grade (grade I, grade II, grade III, and grade IV), bone metastasis (yes or no), liver metastasis (yes or no), brain metastasis (yes or no), primary site (cardia, fundus, body, gastric antrum, lesser, greater, other), radiotherapy (yes or no), chemotherapy (yes or no), surgery (yes or no), marital status (yes or no), and insurance (yes or no). OS and CSS were considered endpoint times. OS and CSS were, respectively, defined as the time from diagnosis to death from all causes and the time from cancer diagnosis to death.

### 2.3. Statistical Analysis

All statistical analyses were performed using the R software (version 4.0.2). *P* value <0.05 (both sides) was considered statistically significant. We obtained relevant prognostic factors through univariate Cox regression analysis and obtained independent prognostic factors through multivariate Cox regression analysis on the basis of univariate Cox regression analysis. The prognostic nomograms for OS and CSS were created separately using the “rms” package, according to the independent prognostic factors. In addition, ROC curves for the prognostic nomograms were established. The area under the curve (AUC) was used to evaluate the discriminative power of the nomograms. In addition, calibration curves and decision curve analysis (DCA) for nomograms were established. Finally, we divided all patients into high- and low-risk groups according to the median risk score and tested the prognostic value of the nomograms using Kaplan-Meier (K-M) analysis.

## 3. Results

### 3.1. Flowchart

**Figure 1:**
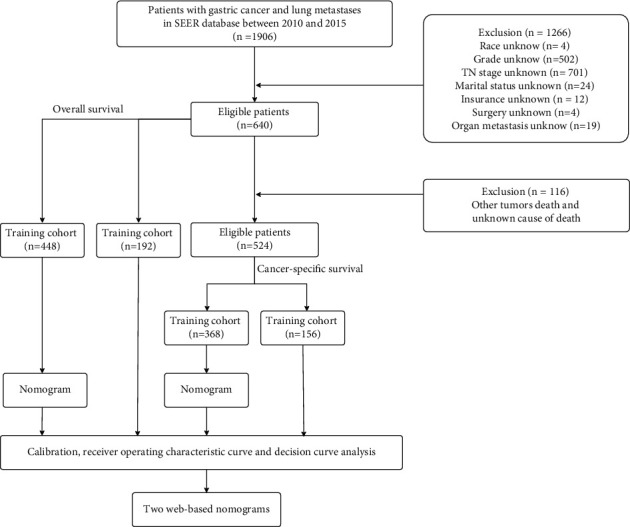
Detailed workflow of study design and analysis.

A detailed workflow is shown in Figure 1.

### 3.2. Characteristics of the Study Population

For the OS analysis, a total of 640 patients were included, 448 patients in the training cohort and the remaining 192 patients in the validation cohort. Among the 640 patients, the number of male patients (69.69%) was higher than that of the female patients (30.31%). A total of 484 patients (75.63%) were white, 75 patients (11.72%) were black, and 81 patients (12.65%) were classified as “other.” Of these patients, 219 were below 60 years of age and 421 were 60 years old or older. The baseline clinicopathological characteristics of patients in the OS group are shown in Table 1.

A total of 524 patients for the CSS analysis were enrolled; 368 patients were included in the training cohort, and the remaining 156 patients were included in the validation cohort. Of the 524 patients, 69.08% were male and 30.92% were female patients. Most of the patients (70.05%) were classified as white. Finally, 197 patients were below 60 years of age, and 327 patients were 60 years old or older. The baseline clinical pathological characteristics of patients in the CSS group are shown in Table 2.

### 3.3. Prognostic Factors for Patients with GCLM

For grouping status of OS, the detailed information of patients with GCLM in the OS group is shown in Table 3. Univariate Cox regression analysis demonstrated that grade II, liver metastasis, radiotherapy, and chemotherapy were OS-related prognostic factors. Multivariate Cox regression analysis showed that grade I1l (*P* value = 0.018, hazard ratios (HR) = 1.896, 95% confidence interval (CI) = 1.118–3.214), liver metastasis (*P* value <0.001, HR = 1.440, 95% CI = 1.179–1.760), and chemotherapy (*P* value <0.001, HR = 0.292, 95% CI = 0.235–0.363) were independent prognostic factors in patients with GCLM.

For grouping status of CSS, more details of the patients with GCLM in the CSS group are listed in Table 4. Univariate Cox regression analysis revealed that race, T2, liver metastasis, primary site, chemotherapy, and marital status were CSS-related prognostic factors. Multivariate COX regression analysis revealed that liver metastasis (*P* value <0.001, HR = 1.524, 95% CI = 1.217–1.909), primary site (greater, *P* value = 0.001, HR = 2.315, 95% CI = 1.395–3.814), chemotherapy (*P* value <0.001, HR = 0.398, 95% CI = 0.317–0.501), and marital status (*P* value = 0.039, HR = 0.778, 95% CI = 0.629–0.988) were independent prognostic factors for GCLM.

### 3.4. Establishment of Nomogram

**Figure 2:**
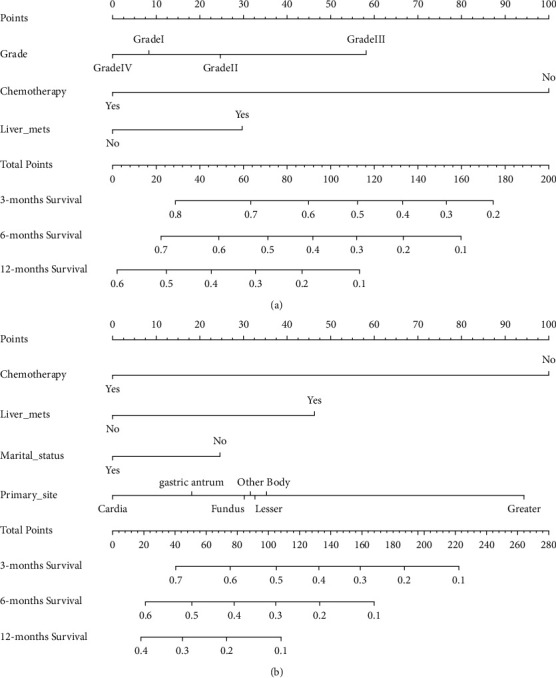
Nomogram. (a) Overall survival (OS) nomogram; (b) cancer-specific survival (CSS) nomogram. OS, overall survival; CSS, cancer-specific survival.

Prognostic nomograms of OS were established according to three independent prognostic factors (Figure 2(a)). Prognostic nomograms of CSS were created according to four independent prognostic factors (Figure 2(b)).

### 3.5. Verification of Nomogram

**Figure 3:**
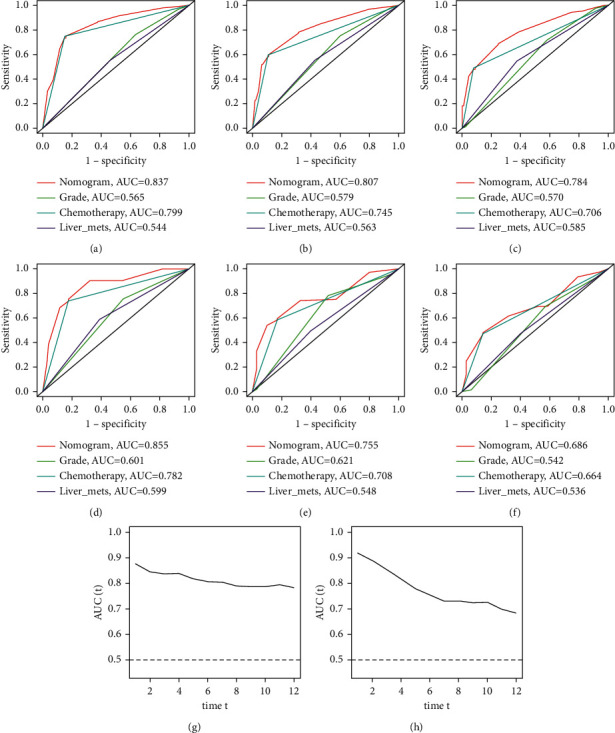
Receiver operating characteristic (ROC) curves of OS. (a–c) ROC curves corresponding to 3, 6, and 12months in the training cohort, respectively; (d–f) ROC curves corresponding to 3, 6, and 12 months in the verification cohort, respectively; (g) the time-dependent ROC curve corresponding to 1 to 12 months in the verification cohort in the training cohort; 3 h, the time-dependent ROC curve corresponding to 1 to 12 months in the verification cohort. ROC, receiver operating characteristic; OS, overall survival.

**Figure 4:**
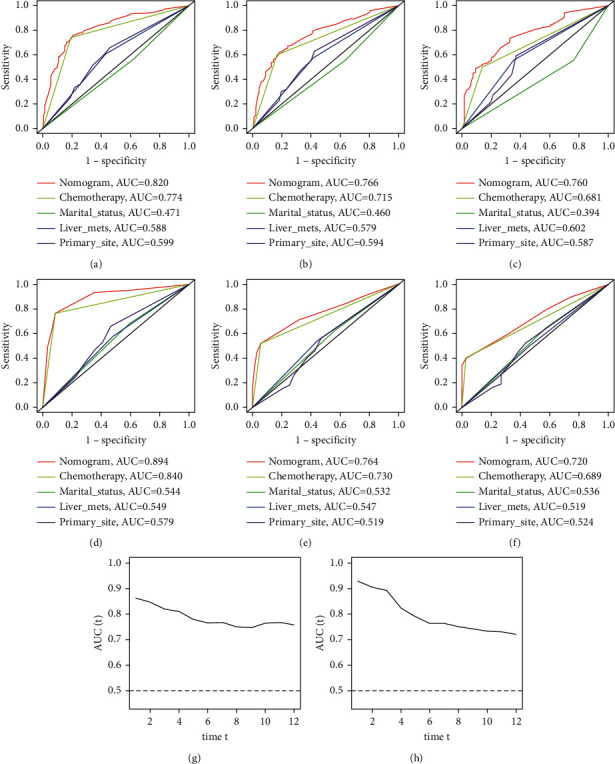
Receiver operating characteristic (ROC) curves of cancer-specific survival (CSS). (a–c) ROC curves corresponding to 3, 6, and 12 months in the training cohort, respectively; (d–f) ROC curves corresponding to 3, 6, and 12 months in the verification cohort, respectively; (g) the time-dependent ROC curve corresponding to 1 to 12 months in the verification cohort in the training cohort; (h) the time-dependent ROC curve corresponding to 1 to 12 months in the verification cohort. ROC, receiver operating characteristic; CSS, cancer-specific survival.

**Figure 5:**
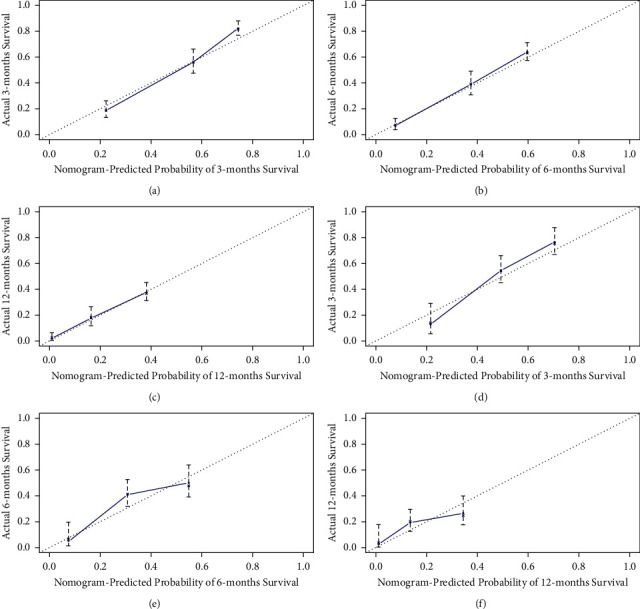
Calibration curves of overall survival (OS). (a–c) Calibration curves corresponding to 3, 6, and 12 months in the training cohort, respectively; (d–f) calibration curves corresponding to 3, 6, and 12 months in the verification cohort, respectively. OS, overall survival.

**Figure 6:**
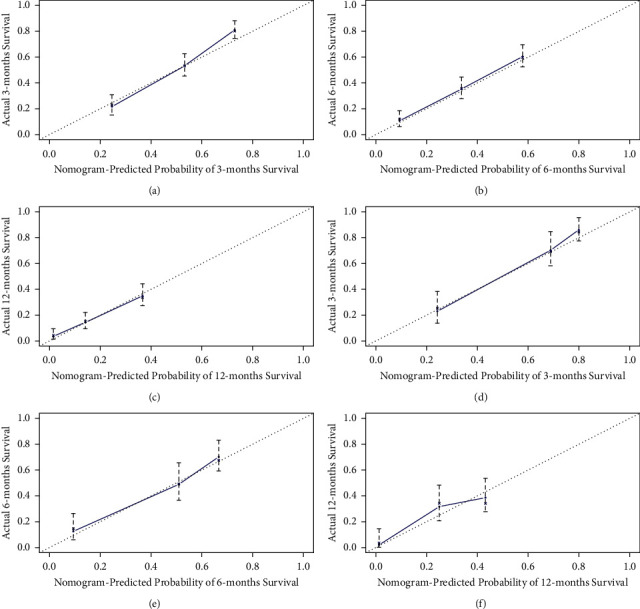
Calibration curves of cancer-specific survival (CSS). (a–c) Calibration curves corresponding to 3, 6, and 12 months in the training cohort, respectively; (d–f) calibration curves corresponding to 3, 6, and 12 months in the verification cohort, respectively. CSS, cancer-specific survival.

**Figure 7:**
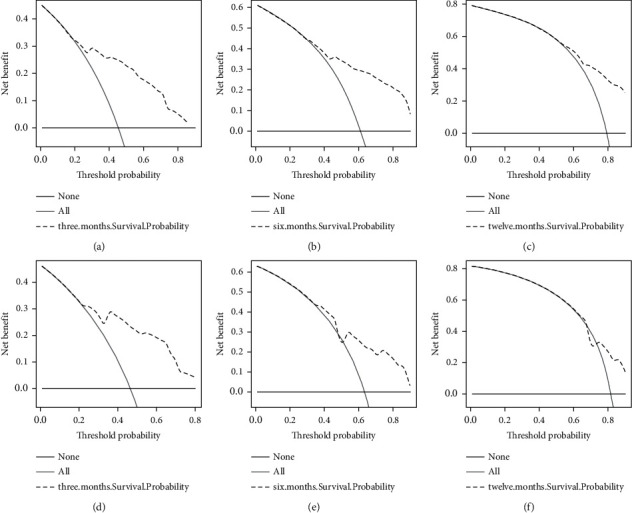
Decision curve analysis (DCA) curves of overall survival (OS). (a–c) DCA corresponding to 3, 6, and 12 months in the training cohort, respectively; (d–f) DCA corresponding to 3, 6, and 12 months in the verification cohort, respectively. DCA, decision curve analysis; OS, overall survival.

**Figure 8:**
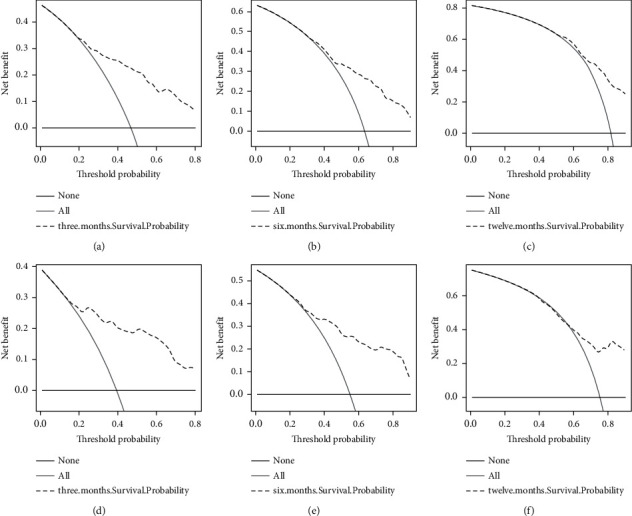
Decision curve analysis (DCA) curves of cancer-specific survival (CSS). (a–c) DCA corresponding to 3, 6, and 12 months in the training cohort, respectively; (d–f) DCA corresponding to 3, 6, and 12 months in the verification cohort, respectively. DCA, decision curve analysis; CSS, cancer-specific survival.

**Figure 9:**
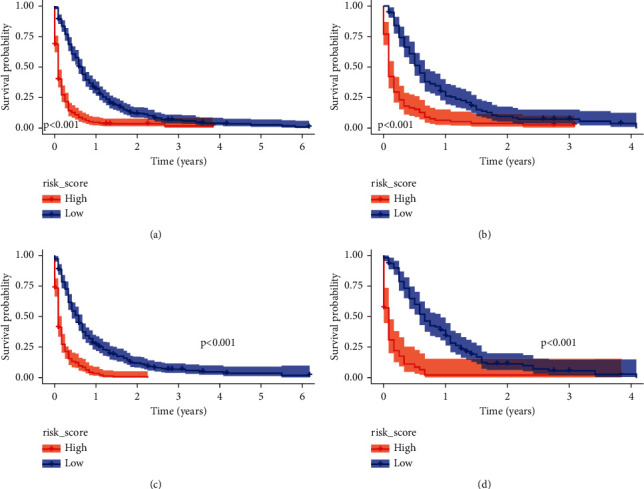
Kaplan-Meier (K-M) survival curves. (a) K-M survival curves in training cohort for OS of GCLM; (b) K-M survival curves in verification queue for OS of GCLM; (c) K-M survival curves in training cohort for CSS of GCLM; (d) K-M survival curves in verification cohort for CSS of GCLM. K-M, Kaplan-Meier; OS, overall survival; CSS, cancer-specific survival.

- ROC of OS: The AUCs at 3, 6, and 12 months were 0.753, 0.799, and 0.732, respectively, in the training cohort (Figures 3(a)–3(c)). In the validation cohort, the AUCs at 3, 6, and 12 months were 0.855, 0.755, and 0.686, respectively (Figures 3(d)–3(f)). The time-dependent ROC curves revealed that the AUC value fluctuated at approximately 0.8 from one month to 12 months (Figures 3(g) and 3(h)).
- ROC of CSS: The AUCs at 3, 6, and 12 months were, respectively, 0.820, 0.766, and 0.760, respectively, in the training cohort (Figures 4(a)–4(c)). The AUCs at 3, 6, and 12 months were separately 0.894, 0.764, and 0.720, respectively, in the validation cohort (Figures 4(d)–4(f)). The time-dependent ROC curves also demonstrated that the AUC value fluctuated at approximately 0.8 from one month to 12 months (Figures 4(g) and 4(h)).
- Calibration curves: The calibration curves at 3, 6, and 12 months for the OS probabilities were in good correspondence with the OS predicted with the nomograms to the actual results (Figures 5(a)–5(f)). The calibration curves for the CSS probabilities at 3, 6, and 12 months also suggested the same better consistency among the CSS forecasted with the nomogram and the actual results (Figures 6(a)–6(f)).
- DCA curves: DCA curves confirmed that nomograms can better predict OS (Figures 7(a)–7(f)) and CSS (Figures 8(a)–8(f)) in patients with GCLM. In addition, K-M survival curves revealed that, for OS (Figures 9(a) and 9(b)) and CSS (Figures 9(c) and 9(d)), patients from the higher risk group had a more unfavorable prognosis than those from the lower risk group.

### 3.6. Establishment of Two Web-Based Nomograms

**Figure 10:**
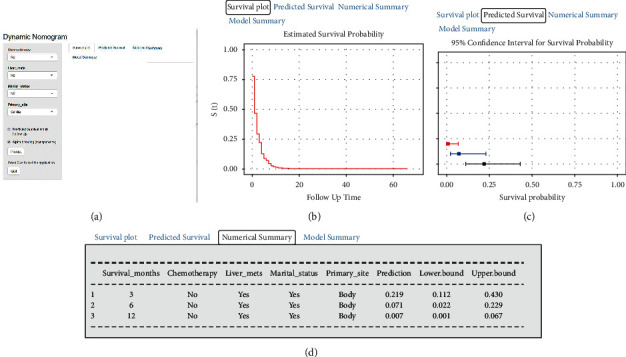
Web-based nomogram. (a) Operation page of web-based nomogram; (b) survival curve of the corresponding patient; (c) survival rates and 95% confidence intervals at 3 months (black line), 6 months (blue line), and 12 months (red line); (d) the prediction accuracy of the corresponding patient.

Based on the above results, we constructed a probabilistic calculator OS (https://zhenghh.shinyapps.io/aclmos/) and CSS (https://zhenghh.shinyapps.io/aslmcss/) based on a dynamic network, which predicts the OS and CSS of patients with GCLM based on previous nomograms (Figure 10(a)). For example, the CSS of a patient with GCLM, who is a married woman with liver metastases, occurs in the gastric body and without chemotherapy. The survival curve of this patient is shown in Figure 10(b). Survival rates and 95% confidence intervals at three months (Figure 10(c), black line), six months (Figure 10(c), blue line), and 12 months (Figure 10(c), red line) can also be observed at the operation interface. In addition, specific numbers are summarized to improve the prediction accuracy (Figure 10(d)). The OS of patients with GCLM can be predicted in the same way.

## 4. Discussion

GC is a malignant tumor of the gastrointestinal tract with a low early diagnosis rate, low surgical resection rate, and high mortality rate [23]. The majority of patients with GC are in the advanced stage at the time of consultation, and 32.6% have distant metastases [24]. Interestingly, the incidence of LM is 14.9% [24]. LM typically indicates advanced tumors, and when not detected and treated in time, the prognosis is extremely poor. In our study, we created two nomograms to predict the prognosis of patients with GCLM. These two nomograms performed well in predicting OS and CSS in patients with GCLM, allowing more precise individualized clinical decision-making and surveillance. Finally, we built two web-based nomograms based on the nomograms. This prediction model can facilitate the prediction of the survival probability of patients with GCLM at a specific time. Clinicians can also arrange personalized treatment plans based on the prediction results.

As we know, survival statistics of GCLM are not optimistic. Therefore, clinicians can identify the risk and protective factors of GCLM, which can result in a good prognosis for patients with GCLM. A number of potential biomarkers that are involved in cadherin-catenin interaction, integrin signaling, and cancer stem cell identification in gastrointestinal cancers have been observed [25]. However, these biomarkers are difficult to measure, have low sensitivity, are expensive, and have few clinical applications. Therefore, it is necessary to actively identify other clinical features related to prognosis in patients with advanced GCLM. In 2019, Wenjie et al. [26] found that age, race, primary site, T stage, and N stage are independently related to CSS in patients with lymph node-positive GC. Studies have shown that the fat content in high muscle tissue is associated with CSS in patients with locally advanced GC [27]. However, so far, few studies have focused on GCLM, and no corresponding nomogram has been established to assess the survival and prognosis of these patients. Previous studies have confirmed that the prognostic factors of liver cancer are quite different from those of liver cancer with bone metastasis [28–31]. Therefore, it is not possible to evaluate the survival of patients with GCLM solely through the prognostic factors of GC, due to possible biases and errors. In this study, we screened the relevant independent prognostic factors of patients with GCLM. More meaningfully, this study integrates these multiple prognostic factors and visual graphs to predict the survival of patients with GCLM through nomograms, which is a practical tool widely used in oncology [32]. The web-based nomograms were based on further upgraded results.

We found that liver metastasis is an independent risk factor for OS and CSS in patients with GCLM. There are two possible reasons for this. First, the liver contains a rich blood supply, tumor metastasis is rapid, patients are already advanced when symptoms appear, and most of them miss the time of surgery. Second, for patients with GCLM and hepatocellular carcinoma (HCC), the prognosis is worse because the patients are lethargic and weak, and their immunity is reduced, typically when they develop complications associated with advanced HCC (such as jaundice, ascites, peritonitis, and hepatic encephalopathy). Chemotherapy was found to be an independent protective factor for OS and CSS. This result confirmed the importance and necessity of chemotherapy in patients with GCLM. The National Comprehensive Cancer Network guidelines clearly state that chemotherapy is recommended for the treatment of patients with unresectable or metastatic GC [33]. A study reported median OS times of 8.6 and 7.9 months for patients with advanced GC treated with cisplatin combined with S-1 (CS) versus cisplatin combined with 5-FU (CF) regimens, respectively (*P*=0.02) [34]. Standardized chemotherapy not only relieves the patients' clinical symptoms but also prolongs the survival time. Hence, it is worthwhile to focus on the possibility of liver metastasis in patients with GCLM. To obtain an excellent prognosis, doctors could prefer chemotherapy for the clinical treatment of patients with GCLM. In addition, we incorporated marital status into our study. The results of this study showed that married patients with GCLM had better clinical prognosis than those who were unmarried. It has been shown that marriage plays a humanistic role during the treatment of oncology patients and that care plays a crucial role in influencing tumor progression [35].

However, there are some limitations to our study. First, although we have set strictly incorporated exclusion standards, the deletion of patients is missing and may cause statistical bias. Second, there is no detailed treatment information in the SEER database, such as specific chemotherapy modalities and surgical procedures. Third, the SEER database has limited coverage, and some important factors such as smoking, alcohol consumption, family history of tumor, and other factors that may affect patient prognosis were not assessed.

## 5. Conclusions

In conclusion, this study revealed that grade, liver metastasis, and chemotherapy were independent prognostic factors for OS, where the risk factors were grade and liver metastasis, and chemotherapy was a protective factor. Liver metastasis, primary site, chemotherapy, and marital status were independent prognostic factors for CSS, where liver metastasis and primary site were risk factors, and chemotherapy and marital status were protective factors. We created two easy-to-use visual web-based nomograms with several clinical and pathological factors to quantitatively predict OS and CSS in patients with GCLM. Moreover, our model may help physicians develop individualized postoperative follow-up strategies.

## Figures and Tables

**Table 1:** Baseline data of clinicopathological characteristics of patients with GCLM in OS group.

Variables	Total cohort (*N* = 640)		Training cohort (*N* = 448)		Validation cohort (*N* = 192)	
	*n*	%	*n*	%	*n*	%
Age						
<60	219	34.2	158	35.3	61	31.8
≥60	421	65.8	290	64.7	131	68.2
Race						
Black	75	11.7	53	11.8	22	11.5
Other	81	12.7	51	11.4	30	15.6
White	484	75.6	344	76.8	140	72.9
Sex						
Female	194	30.3	127	28.3	67	34.9
Male	446	69.7	321	71.7	125	65.1
Histologic type						
Adenocarcinoma	407	63.6	288	64.3	119	62.0
Signet ring cell	94	14.7	66	14.7	28	14.6
Intestinal type	42	6.6	26	5.8	16	8.3
Other	97	15.2	68	15.2	29	15.1
T stage						
T1	252	39.4	168	37.5	84	43.8
T2	40	6.3	28	6.3	12	6.3
T3	147	23	106	23.7	41	21.4
T4	201	31.4	146	32.6	55	28.6
N stage						
N0	262	40.9	185	41.3	77	40.1
N1	292	45.6	198	44.2	94	49.0
N2	43	6.7	35	7.8	8	4.2
N3	43	6.7	30	6.7	13	6.8
Grade						
Grade I	26	4.1	16	3.6	10	5.2
Grade II	178	27.8	124	27.7	54	28.1
Grade III	426	66.6	302	67.4	124	64.6
Grade IV	10	1.6	6	1.3	4	2.1
Bone metastasis						
No	526	82.2	372	83	154	80.2
Yes	114	17.8	76	17	38	19.8
Liver metastasis						
No	325	50.8	222	49.6	103	53.6
Yes	315	49.2	226	50.4	89	46.4
Brain metastasis						
No	622	97.2	436	97.3	186	96.9
Yes	18	2.8	12	2.7	6	3.1
Primary site						
Cardia	291	45.5	201	44.9	90	46.9
Fundus	35	5.5	22	4.9	13	6.8
Body	44	6.9	31	6.9	13	6.8
Gastric antrum	74	11.6	53	11.8	21	10.9
Lesser	28	4.4	21	4.7	7	3.6
Greater	30	4.7	22	4.9	8	4.2
Other	138	21.6	98	21.9	40	20.8
Radiotherapy						
No	492	76.9	348	77.7	144	75.0
Yes	148	23.1	100	22.3	48	25.0
Chemotherapy						
No	256	40	179	40	77	40.1
Yes	384	60	269	60	115	59.9
Surgery						
No	580	90.6	407	90.8	173	90.1
Yes	60	9.4	41	9.2	19	9.9
Marital status						
No	255	39.8	182	40.6	73	38.0
Yes	385	60.2	266	59.4	119	62.0
Insurance						
No	37	5.8	30	6.7	7	3.6
Yes	603	94.2	418	93.3	185	96.4

**Table 2:** Baseline data of clinicopathological characteristics of patients with GCLM in CSS group.

Variables	Total cohort (*N* = 524)		Training cohort (*N* = 368)		Validation cohort (*N* = 156)	
	*n*	%	*n*	%	*n*	%
Age						
<60	197	37.6	131	35.6	66	42.3
≥60	327	62.4	237	64.4	90	57.7
Race						
Black	67	12.8	50	13.6	17	10.9
Other	69	13.2	49	13.3	20	12.8
White	388	74	269	73.1	119	76.3
Sex						
Female	162	30.9	109	29.6	53	34.0
Male	362	69.1	259	70.4	103	66.0
Histologic type						
Adenocarcinoma	340	64.9	247	67.1	93	59.6
Signet ring cell	76	14.5	46	12.5	30	19.2
Intestinal type	36	6.9	26	7.1	10	6.4
Other	72	13.7	49	13.3	23	14.7
T stage						
T1	209	39.9	148	40.2	61	39.1
T2	36	6.9	27	7.3	9	5.8
T3	117	22.3	84	22.8	33	21.2
T4	162	30.9	109	29.6	53	34.0
N stage						
N0	207	39.5	144	39.1	63	40.4
N1	254	48.5	187	50.8	67	42.9
N2	26	5	14	3.8	12	7.7
N3	37	7.1	23	6.3	14	9.0
Grade						
Grade I	19	3.6	15	4.1	4	2.6
Grade II	147	28.1	105	28.5	42	26.9
Grade III	349	66.6	242	65.8	107	68.6
Grade IV	9	1.7	6	1.6	3	1.9
Bone metastasis						
No	432	97.1	303	82.3	129	82.7
Yes	92	2.9	65	17.7	27	17.3
Liver metastasis						
No	257	49	179	48.6	78	50.0
Yes	267	51	189	51.4	78	50.0
Brain metastasis						
No	509	97.1	356	96.7	153	98.1
Yes	15	2.9	12	3.3	3	1.9
Primary site						
Cardia	240	45.8	164	44.6	76	48.7
Fundus	33	6.3	22	6	11	7.1
Body	38	7.3	29	7.9	9	5.8
Gastric antrum	63	12	46	12.5	17	10.9
Lesser	20	3.8	11	3	9	5.8
Greater	24	4.6	19	5.2	5	3.2
Other	106	20.2	77	20.9	29	18.6
Radiotherapy						
No	401	76.5	276	75	125	80.1
Yes	123	23.5	92	25	31	19.9
Chemotherapy						
No	205	39.1	158	42.9	47	30.1
Yes	319	60.9	210	57.1	109	69.9
Surgery						
No	481	91.8	340	92.4	141	90.4
Yes	43	8.2	28	7.6	15	9.6
Marital status						
No	209	39.9	152	41.3	57	36.5
Yes	315	60.1	216	58.7	99	63.5
Insurance						
No	31	5.9	22	6	9	5.8
Yes	493	94.1	346	94	147	94.2

**Table 3:** Univariate and multivariate Cox proportional hazards regression analysis of patients with GCLM in the OS group.

Variables	Univariate Cox regression analysis		Multivariate Cox regression analysis	
	HR (95% CI)	*P*	HR (95% CI)	*P*
Age				
<60	Reference			
≥60	1.044 (0.854–1.277)	0.671		
Race				
Black	Reference			
Other	0.979 (0.658–1.457)	0.918		
White	0.788 (0.586–1.061)	0.117		
Sex				
Female	Reference			
Male	0.99 (0.799–1.225)	0.923		
Histologic type				
Adenocarcinoma	Reference			
Signet ring cell	1.044 (0.786–1.388)	0.765		
Intestinal type	1.358 (0.901–2.047)	0.144		
Other	1.144 (0.873–1.498)	0.33		
T stage				
T1	Reference			
T2	0.705 (0.459–1.083)	0.11		
T3	0.944 (0.735–1.214)	0.656		
T4	1.219 (0.969–1.535)	0.091		
N stage				
N0	Reference			
N1	1.009 (0.820–1.243)	0.929		
N2	0.961 (0.664–1.391)	0.833		
N3	1.166 (0.790–1.721)	0.44		
Grade				
Grade I	Reference		Reference	
Grade II	1.458 (0.850–2.501)	0.171	1.275 (0.738–2.201)	0.383
Grade III	1.864 (1.105–3.144)	0.02	1.896 (1.118–3.214)	0.018
Grade IV	1.238 (0.410–3.743)	0.705	0.942 (0.310–2.864)	0.916
Bone metastasis				
No	Reference			
Yes	1.075 (0.833–1.388)	0.58		
Liver metastasis				
No	Reference			
Yes	1.309 (1.080–1.587)	0.006	1.440 (1.179–1.760)	<0.001
Brain metastasis				
No	Reference			
Yes	1.434 (0.806–2.550)	0.22		
Primary site				
Cardia	Reference			
Fundus	1.487 (0.956–2.315)	0.079		
Body	1.179 (0.792–1.754)	0.418		
Gastric antrum	1.224 (0.893–1.676)	0.208		
Lesser	1.211 (0.764–1.922)	0.416		
Greater	1.182 (0.737–1.895)	0.488		
Other	1.28 (0.995–1.646)	0.054		
Radiotherapy				
No	Reference			
Yes	0.761 (0.606–0.955)	0.019	0.979 (0.770–1.244)	0.859
Chemotherapy				
No	Reference			
Yes	0.312 (0.253–0.384)	<0.001	0.292 (0.235–0.363)	<0.001
Surgery				
No	Reference			
Yes	0.835 (0.598–1.167)	0.291		
Marital status				
No	Reference			
Yes	0.844 (0.694–1.025)	0.087		
Insurance				
No	Reference			
Yes	1.076 (0.723–1.602)	0.718		

**Table 4:** Univariate and multivariate Cox proportional hazards regression analysis of patients with GCLM in the CSS group.

Variables	Univariate Cox regression analysis		Multivariate Cox regression analysis	
	HR (95% CI)	*P*	HR (95% CI)	*P*
Age				
<60	Reference			
≥60	0.945 (0.759–1.176)	0.612		
Race				
Black	Reference		Reference	
Other	0.823 (0.549–1.232)	0.343	0.916 (0.602–1.393)	0.681
White	0.707 (0.519–0.963)	0.028	0.883 (0.635–1.227)	0.458
Sex				
Female	Reference			
Male	0.976 (0.775–1.230)	0.837		
Histologic type				
Adenocarcinoma	Reference			
Signet ring cell	1.064 (0.763–1.484)	0.715		
Intestinal type	1.207 (0.798–1.825)	0.372		
Other	1.152 (0.846–1.570)	0.368		
T stage				
T1	Reference		Reference	
T2	0.594 (0.378–0.932)	0.024	0.71 (0.446–1.131)	0.149
T3	0.854 (0.648–1.124)	0.26	1.121 (0.840–1.497)	0.436
T4	1.088 (0.844–1.402)	0.513	1.15 (0.882–1.498)	0.302
N stage				
N0	Reference			
N1	0.922 (0.737–1.153)	0.475		
N2	0.864 (0.498–1.500)	0.603		
N3	0.865 (0.551–1.358)	0.528		
Grade				
Grade I	Reference			
Grade II	1.282 (0.732–2.245)	0.385		
Grade III	1.632 (0.950–2.806)	0.076		
Grade IV	2.121 (0.813–5.530)	0.124		
Bone metastasis				
No	Reference			
Yes		0.146		
Liver metastasis				
No	Reference		Reference	
Yes	1.47 (1.186–1.821)	<0.001	1.524 (1.217–1.909)	<0.001
Brain metastasis				
No	Reference			
Yes	1.114 (0.610–2.033)	0.725		
Primary site				
Cardia	Reference		Reference	
Fundus	1.593 (1.006–2.522)	0.047	1.312 (0.824–2.091)	0.253
Body	1.337 (0.880–2.030)	0.173	1.364 (0.882–2.108)	0.163
Gastric antrum	1.498 (1.067–2.105)	0.02	1.206 (0.846–1.718)	0.301
Lesser	1.185 (0.642–2.189)	0.587	1.409 (0.732–2.710)	0.305
Greater	2.024 (1.238–3.308)	0.005	2.315 (1.395–3.841)	0.001
Other	1.367 (1.035–1.805)	0.028	1.26 (0.932–1.704)	0.133
Radiotherapy				
No	Reference			
Yes	0.813 (0.640–1.033)	0.091		
Chemotherapy				
No	Reference		Reference	
Yes	0.388 (0.311–0.484)	<0.001	0.398 (0.317–0.501)	<0.001
Surgery				
No	Reference			
Yes	0.715 (0.474–1.077)	0.109		
Marital status				
No	Reference		Reference	
Yes	0.735 (0.592–0.912)	0.005	0.788 (0.629–0.988)	0.039
Insurance				
No	Reference			
Yes	0.954 (0.600–1.518)	0.843		

## Data Availability

The dataset from the SEER database which was generated and/or analyzed during the current study is available in the SEER dataset repository (https://seer.cancer.gov/).

## Abbreviations

AUC: Area under the curve
CSS: Cancer-specific survival
DCA: Decision curve analysis
GC: Gastric cancer
GCLM: Gastric cancer and lung metastasis
LM: Lung metastasis
OS: Overall survival
ROC: Receiver operating characteristic
SEER: Surveillance, Epidemiology, and End Results.
